# *IL2RA* Methylation and Gene Expression in Relation to the Multiple Sclerosis-Associated Gene Variant rs2104286 and Soluble IL-2Rα in CD8^+^ T Cells

**DOI:** 10.3389/fimmu.2021.676141

**Published:** 2021-07-27

**Authors:** Sophie Buhelt, Hannah-Marie Laigaard, Marina Rode von Essen, Henrik Ullum, Annette Oturai, Finn Sellebjerg, Helle Bach Søndergaard

**Affiliations:** ^1^Danish Multiple Sclerosis Center, Department of Neurology, Copenhagen University Hospital, Rigshospitalet, Glostrup, Denmark; ^2^Statens Serum Institut, Copenhagen, Denmark; ^3^Department of Clinical Medicine, University of Copenhagen, Copenhagen, Denmark

**Keywords:** multiple sclerosis, CD8^+^ T cells, interleukin-2 receptor alpha, sCD25, *IL2RA*, rs2104286, IL-7 receptor alpha, allele specific methylation

## Abstract

CD8^+^ T cells are involved in the pathogenesis of multiple sclerosis (MS). The interleukin-2 receptor α (IL-2Rα) is important for CD8^+^ T cell function, and single nucleotide polymorphisms (SNPs) in the *IL2RA* gene encoding IL-2Rα increase the risk of MS. Therefore, in isolated CD8^+^ T cells we investigated *IL2RA* gene methylation and gene expression in relation to the MS-associated *IL2RA* SNP rs2104286 and soluble IL-2Rα (sIL-2Rα). We have identified allele specific methylation of the CpG-site located in intron 1 that is perturbed by the rs2104286 SNP in CD8^+^ T cells from genotype-selected healthy subjects (HS). However, methylation of selected CpG-sites in the promotor or 5’UTR region of the *IL2RA* gene was neither associated with the rs2104286 SNP nor significantly correlated with *IL2RA* gene expression in HS. In CD8^+^ T cells from HS, we explored expression of immune relevant genes but observed only few associations with the rs2104286 SNP. However, we found that sIL-2Rα correlated negatively with expression of 55 immune relevant genes, including the IL-7 receptor gene, with Spearman’s rho between -0.49 and -0.32. Additionally, in HS by use of flow cytometry we observed that the IL-7 receptor on naïve CD8^+^ T cells correlated negatively with sIL-2Rα and was downregulated in carriers of the rs2104286 MS-associated risk genotype. Collectively, our study of resting CD8^+^ T cells indicates that the rs2104286 SNP has a minor effect and sIL-2Rα may negatively regulate the CD8^+^ T cell response.

## Introduction

Multiple sclerosis (MS) is an immune-mediated disease of the central nervous system (CNS) where peripherally activated immune cells cross the blood brain barrier and initiate an inflammatory process within the CNS that damages myelin, oligodendrocytes and axons leading to neurological dysfunction (1).

Observations in human samples implicate a role of CD8^+^ T cells in MS disease development and progression (2, 3). Differences in gene expression of peripheral blood mononuclear cells (PBMC) between MS patients and healthy subjects (HS) can in part be tracked to differences in gene expression in CD8^+^ T cells (4). In MS patients, CD8^+^ T cells outnumber CD4^+^ T cells in all brain lesion types independent of disease course, severity and duration (5–8). In lesions, the majority of CD8^+^ T cells are oligoclonally expanded (9, 10), enriched for a cytotoxic effector memory phenotype and express markers associated with homing, activation and proliferation (7, 8, 11).

Genetic studies decoding the complex genetic heritage of MS have established that the single nucleotide polymorphism (SNP) rs2104286 in the first intron of the *IL2RA* gene increases the risk of developing multiple sclerosis (12). In both CD4^+^ and CD8^+^ T cells, expression of the *IL2RA* gene is increased in MS patients compared with HS and in T cells a specific CpG-site located in the *IL2RA* 5’UTR is hypomethylated in MS patients compared to HS coherent with an increased *IL2RA* expression in MS patients (13). The *IL2RA* gene encodes the unique α-chain of the trimeric interleukin-2 (IL-2) receptor (IL-2Rα, CD25) (14). Cell surface bound IL-2Rα confers high-affinity binding and is needed for optimal IL-2 signalling (15–17). Consequently, heterogeneity in IL-2Rα expression determines a cells response to IL-2 (16, 17).

Homozygous risk allele carriers (T) for the MS-associated SNP rs2104286 have reduced STAT5 phosphorylation (18), higher expression of the *IL2RA* gene in MS patients and an increased level of soluble IL-2Rα (sIL-2Rα) in both MS and HS (19, 20). Soluble IL-2Rα has been shown to antagonize the IL-2 signal (21, 22). Collectively, these observations indicate that the MS-associated SNP rs2104286 influences IL-2 signalling.

In CD8^+^ T cells, strength and duration of the IL-2 signal influences effector function, proliferation and differentiation (14). Prolonged IL-2 signalling promotes expansion, the differentiation of KLRG^hi^ effector cells and the abundance of proteins and genes associated with effector function such as lymphotoxin-α, perforin and granzymes (16, 23–25). Transient IL-2 signalling favours effector memory differentiation and promotes generation and survival of memory cells capable of a robust recall response (25–28). Notable, several studies of the effect of IL-2 were performed by manipulating the surface expression of IL-2Rα to be either high or low on CD8^+^ T cells (16, 25, 26, 28).

Few studies have investigated association between the MS-associated *IL2RA* SNP and changes in CD8^+^ T cells and only on a protein or cellular level (29, 30). Therefore, in the present study we analysed isolated CD8^+^ T cells to investigate associations between the MS-associated *IL2RA* SNP rs2104286 and methylation of the *IL2RA* gene as well as expression of immune relevant genes. Furthermore, in the isolated CD8^+^ T cells from HS we explored possible correlations between expression of the immune relevant genes and the level of sIL-2Rα.

## Materials and Methods

### Study Population and Ethics

Our study had two separate cohorts: cohort 1 consisting of 40 HS selected based on the MS-associated SNP rs2104286 in the *IL2RA* gene (i.e., genotype-selected) and cohort 2 consisting of 12 HS and 12 patients with RRMS. The majority of hypotheses of the study were explored in HS and primarily in the genotype-selected cohort 1, as results from previous studies have suggested that biological alterations in established MS compromise the ability to study biological changes related to SNPs associated with risk of developing MS (19, 21).

In cohort 1, the 40 HS were selected from 1000 healthy blood donors previously included in the Danish Multiple Sclerosis Center’s contribution to the International Multiple Sclerosis Genetics Consortiums (IMSGC) replication chip study (31). The 40 HS were equally distributed between homozygous carriers of the risk allele (T; mean age 44 years, range 24–66) and homozygous carriers of the protective allele (C; mean age 46 years, range 24 - 68) for the MS-associated SNP rs2104286 as well as the MS-associated intergenic SNP rs11256593 near the *IL2RA* gene (31), which is in strong linkage disequilibrium with rs2104286. The sex distribution was the same in both groups. The HS were from the Capital and Zealand region of Denmark and were of European ancestry. They reported no family history of MS and did not have any known autoimmune diseases. All HS were self-reported non-smokers at least 1-year prior to recruitment, as previous studies have found that smoking alters methylation (32). The 40 HS have previously been a part of a published study from our group that analysed associations between surface expression of IL-2Rα on T cells and the MS-associated *IL2RA* SNPs (29).

Cohort 2 was included in the analysis of allele specific methylation and consisted of 12 HS (40.3 years, range 27 - 57) of which two also were included in cohort 1 as well as 12 untreated patients with relapsing-remitting MS (40.3 years, range 25 – 55) diagnosed according to the 2010 revised McDonald criteria (33). Untreated was defined as one month since last steroid treatment and at least 3 months since last immunomodulatory treatment. Patients had not received strong immunosuppressive drugs, e.g. cyclophosphamide or mitoxantrone, or cell-depleting monoclonal antibody therapy. Samples from HS were sex and age matched and collected in the same time period as patients with MS and had no autoimmune, neurological or chronic illness.

Written informed consent was obtained from all participants. The study was approved by the regional ethical committee (KF-01 314009, KF-01114309).

### Sample Collection and PBMC Isolation

Venous blood was sampled in BD vacutainer EDTA tubes (BD Biosciences, Lyngby, Denmark). In cohort 1, participants were fasting at the time of blood sampling. Within 1 hour the venous blood was diluted with phosphate buffered saline (PBS) (Gibco/Life Technologies, CA, USA) containing EDTA (Ambion) and isolation of the PBMC was performed by density gradient centrifugation using Lymphoprep (Axis-Shield, Oslo, Norway) and washed twice in cold PBS/2mM EDTA. PBMC from participants were immediately either subjected to isolation of CD8^+^ T cell populations (cohort 1), flow cytometry analysis (cohort 1) or were cryopreserved and snap frozen (cohort 2).

### Genotyping

Genotyping of cohort 1 was conducted by the IMSGC and performed on Illumina’s MS replication custom bead chip (31). Cohort 2 was genotyped for SNPs rs2104286 and rs11256593 on DNA purified from snap frozen PBMC using the Nucleospin tissue kit (Macherey-Nagel GmbH & Co. KG, Düren, Germany) according to manufacturer’s guidelines. DNA purity and concentration were measured using the Nanodrop 2000 spectrophotometer (Thermo Fischer Scientific, Waltham, MA, USA). SNP genotyping was performed with TaqMan Fast Universal PCR Master Mix and predesigned primers and probes from TaqMan allelic discrimination assays on a ViiA7 instrument all from Thermo Fisher Scientific, followed by genotype scoring as described by the manufacturer. TaqMan genotyping was performed in duplicates and only genotype detection threshold above 97% was accepted.

### CD8^+^ T Cell Isolation

In cohort 1, CD8^+^ T cells were isolated by negative selection from freshly isolated PBMC using the CD8^+^ T Cell Isolation Kit from Miltenyi Biotec (Miltenyi Biotec GmbH, Bergisch Gladbach, Germany) and the Quadro- or MidiMACS separator (Miltenyi Biotec GmbH) in combination with LS columns (Miltenyi Biotec GmbH) according to the manufacturer’s instructions. Subsequently, isolated CD8^+^ T cells were applied to an extra LS column in the Quadro- or MidiMACS separator to increase purity. The majority of isolated CD8^+^ T cells were stabilized with RNAlater (Thermo Fischer Scientific) and stored at -80°C. Approximately 50,000 isolated CD8^+^ T cells were stained for CD3 (BV421; UCHT1; BioLegend, San Diego, CA, USA), CD4 (APC/AF750; s3.5; Life Technologies, Waltham, MA, USA), CD8 (APC; RPA-T8, BioLegend) and Life/Death (BV510; Life Technologies) and purity was evaluated on a FACS Canto II 8 colour flow cytometer (BD Biosciences, San Jose, CA, USA). Mean purity for the isolated CD8^+^ T cells was 91.8% for homozygous risk allele carriers (T) and 91.4% for homozygous protective allele carriers (C).

In cohort 2, cryopreserved PBMC were thawed and stained with fluorochrome-conjugated Ab against CD3 (FITC; UCHT1) and CD8 (APC, HIT8) both from BioLegend. Subsequently, CD3^+^CD8^+^ T cells were isolated by a 4-way fluorescence-activated cell sorting (FACS) from the stained PBMC using a FACS Aria II (BD Biosciences). Directly after, the isolated cells were lysed in Qiazol lysis buffer (Qiagen, Hilden, Germany) and stored at -80°C. Mean purity for the isolated CD8^+^ T cells was 99.5%.

### Flow Cytometry Analysis

Staining procedure and flow cytometry analysis of cohort 1 have previously been described in detail (29). In brief, freshly isolated PBMC were stained with fluorochrome-conjugated antibodies for CD3 (PE/Cy7; UCHT1), CD8 (BV605; RPA-T8), CD45RA (FITC; HI100), CD127/IL-7Rα (BV421; A019D5) and CD197/CCR7 (AF647; G043H7), all from BioLegend, to analyse differentiation and expression of IL-7Rα on CD8^+^ T cells. Matched isotype controls were applied for IL-7Rα, CD45RA and CCR7. FlowJo software (Tree Star, Ashland, OR, USA) was used for data analysis. Staining procedure and data analysis were performed blinded to genotype.

### DNA Purification and Methylation

In cohort 1, DNA for methylation studies was purified using Prep^®^DNA/RNA/miRNA Universal Kit according to manufacturer’s instructions (Qiagen). In cohort 2, DNA extraction was based on the “User-Developed Protocol” provided by Qiagen but several steps were optimized. For the optimized DNA extraction protocol see **Supplementary Method**.

In cohort 1 and 2, DNA methylation was analysed according to manufacturer’s instructions and included the following steps: 1) bisulfite conversion (BSC), 2) PCR amplification and 3) pyrosequencing.

Bisulfite conversion (BSC) of DNA (300 ng) was done using EZ-96 DNA Methylation-Lightning TM Kit (Zymo Research, Irvine, CA, USA) modifying every unmethylated cytosine (C) into uracil and post PCR into thymidine (T) while methylated Cs remain methylated. 100% and 0% methylated DNA (Zymo Research) were included in the BSC and used to generate the 50% methylated DNA control. The C > T converted DNA and controls were used as templates for PCR amplification.

PCR amplification was performed using PyroMark^®^PCR Kit (Qiagen) and 10 μM primer was used in each reaction. Primer pairs were designed using PyroMark AssayDesign 2.0 software (Qiagen) or SnapGene 4.0.8 (GSL Biotech LLC, Chicago, IL, USA). **Supplementary Table 1** shows the unbiased primer pairs used to amplify each stated fragment. The reverse primers were biotinylated to enable isolation of single stranded DNA (ssDNA) prior to the pyrosequencing. gDNA and H_2_O were used as a negative control and amplified with the same primer pairs as each fragment. Bisulfite converted universal methylated human DNA (Zymo Research) was used as a positive control. PCR fragments were tested for correct band-yield using the Agilent 2100 Bioanalyzer capillary electrophoresis and Agilent DNA 1000 Kit (Agilent Technologies, Santa Clara, CA, USA).

Pyrosequencing was performed using a PyroMark^®^Q24 machine (Qiagen). Forward sequencing primers for each fragment were designed using PyroMark AssayDesign 2.0 software (Qiagen) (see **Supplementary Table 1**). Pyrosequencing assays were designed using PyroMarks Q24 2.0.7 software (Qiagen). Shortly, biotin-labelled ssDNA fragments were isolated according to the protocol PyroMark Q24 User Manual (Qiagen) using the Qiagen PyroMark Q24 Work Station and underwent pyrosequencing with 10 μM sequencing primer. The percent methylation for each CpG-site within the target sequence was calculated using PyroMarks Q24 2.0.7 software (Qiagen). To test if the percent methylation output was trustworthy 100%, 50% and 0% methylation DNA standards were purchased (Zymo Research) and pyrosequenced as controls. Additionally, gDNA and H_2_O was pyrosequenced as negative controls.

### RNA Purification and Gene Expression Analysis of CD8^+^ T Cells

In cohort 1, RNA was purified using AllPrep^®^DNA/RNA/miRNA Universal Kit according to manufacturer’s instructions (Qiagen). For multiplex gene expression analysis, the Nanostring nCounter^®^ platform was used. 100 ng total RNA in 7-20 µl (5-14ng/µl) was used as input. Samples were hybridized to target specific barcodes consisting of a capture probe and a reporter probe using the Nanostring nCounter^®^ PanCancer Immune Profiling panel (NanoString Technologies Inc, Seattle, WA, USA). After hybridization, target RNA was isolated by magnetic bead sorting and immobilized by applying an electric current as described in the hybridization protocol in the nCounter XT Assay user manual (NanoString Technologies Inc). Gene expression data were normalized with *CD8A* and *CD8B* that are stably expressed in CD8^+^ T cells (34). Two outliers were excluded after nSolver gene expression analysis, why only 38 genotype-selected HS were analysed in cohort 1.

### Network Analysis

For exploration of biological processes related to the genes identified as significantly associated with sIL-2Rα we used the Functional Annotation Clustering Tool in the open-source Database for Annotation, Visualisation and Integrated Discovery (DAVID) (http://david.abcc.ncifcrf.gov) - a biological module-centric algorithm (35). Data were analysed by high classification stringency and with standard settings. Benjamini-Hochberg was used to adjust for multiple testing in the DAVID Functional Annotation Clustering.

### Measurements of sIL-2Rα

In cohort 1, measurements of sIL-2Rα were performed in serum isolated from the same blood draw used to isolate PBMCs and subsequently CD8^+^ T cells by using a sandwich ELISA (Quantikine R&D systems, Abingdon, United Kingdom) according to manufacturer’s instructions. Plates were read at 450 nm and 540 nm using a Biotek microplate reader (Synergy HT, Holm&Halby, Broendby, Denmark) and the median coefficient of variation (CV) for sIL-2Rα was 7.3%.

### Statistical Analysis

Statistical analyses were performed using IBM SPSS Statistics 22 (IBM, Armonk, NY, USA) where p-value ≤ 0.05 was considered as statistically significant. All data were analysed non-paired. Normal distribution of the data was tested by the Kolmogorov Smirnov and Shapiro Wilk test. In the analysis of methylation in relation to SNP rs2104286 a one-way analysis of variance (ANOVA) or an unpaired t-test were used depending on the number of compared genotype groups. The analysis of differential gene expression in relation to genotype was performed by an analysis of covariance (ANCOVA) and adjusted for sex and age. Differences in frequency of IL-7Rα on CD8^+^ T cells in relation to genotype were analysed by a non-parametric Mann-Whitney U test. Correlations were analysed using non-parametric Spearman rank correlation. All figures were prepared in GraphPad Prism v8 (GraphPad, San Diego, CA, USA).

## Results

### Allele Specific Methylation

**Figure 1:**
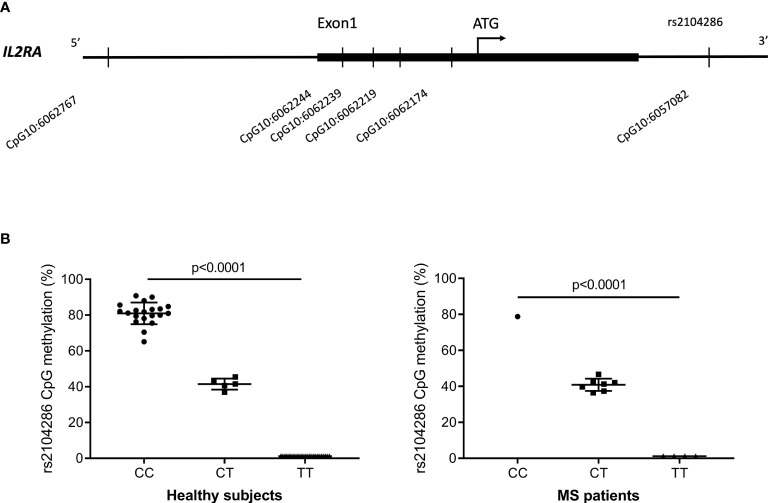
**(A)** Overview of the 6 CpG-sites in the *IL2RA* regulatory region and intron 1 that passed quality control and were methylated above background. Four CpG-sites were in the region corresponding to the 5’UTR and 1 CpG-site was located upstream of this in the promotor region. CpG10:6057082 was located in intron 1 at the site of rs2104286. **(B)** Methylation at the CpG10:6057082 that is perturbed by the MS-associated *IL2RA* rs2104286 SNP in healthy subjects (CC = 20; CT = 5; TT = 25) and MS patients (CC = 1; CT = 7; TT = 4). Of the 50 HS, 38 were from cohort 1 and 12 were from cohort 2. Data were analysed by one-way analysis of variance. Error-bars represent mean with SD.

Methylation status at the CpG10:6057082 (**Figure 1A**) that is perturbed by the rs2104286 SNP was investigated across all three *IL2RA* genotype groups (CC, CT, TT) among 50 HS and 12 MS patients. In the isolated CD8^+^ T cells from HS, as well as MS patients, we observed that homozygous carriers were either highly methylated (protective allele (C) carriers) or not methylated (risk allele (T) carriers) while heterozygous carriers (CT) had an intermediate methylation (**Figure 1B**).

### Promotor Region and 5’UTR Methylation of the *IL2RA* Gene

In the analysis of methylation in the *IL2RA* regulatory region, we selected CpG-sites based on results from two previous studies of the *IL2RA* gene in relation to disease status for either MS or type 1 diabetes (T1D) (13, 36). In total 10 CpG-sites were analysed in two amplicons and of these, we continued with one CpG-site in the promoter region and four CpG-sites in the 5’UTR region that passed quality control and were methylated at levels above background (**Supplementary Table 2** and **Figure 1A**).

In the 40 genotype-selected HS, none of the five CpG-sites were significantly associated with the MS-associated *IL2RA* genotype (**Supplementary Figure 1**). Furthermore, *IL2RA* gene expression showed no correlation with methylation at the five CpG-sites in CD8^+^ T cells (data not shown).

### Gene Expression in Relation to the MS-Associated *IL2RA* Genotype

In CD8^+^ T cells from 38 genotype-selected HS, we observed that only 482 of the 783 immune relevant genes were expressed above quantification threshold in ≥ 50% of the analysed samples. Comparing healthy homozygote carriers of the risk (T) and protective allele (C) for the MS-associated SNP rs2104286 in the *IL2RA* gene, only 3 of the 482 genes expressions were associated with the rs2104286 SNP with a p-value ≤ 0.05 when adjusted for sex and age. None of the genes had FDR q-values ≤ 0.05. *GZMH* and *LILRB1* were expressed at higher levels in TT-carriers and *LRRN3* was expressed at lower levels in TT-carriers (1.58, 1.56 and -1.55 log2 fold change, respectively). No change in *IL2RA* expression was observed between the genotype groups.

### Gene Expression in Relation to sIL-2Rα

In the 40 genotype-selected HS, we confirmed the well-known association between the level of sIL-2Rα and rs2104286 (data not shown) (20). In a previous study from our group, gene expression in PBMC correlated with the concentration of sIL-2Rα in HS (19). Therefore, we investigated if gene expression in CD8^+^ T cells correlated with sIL-2Rα. In CD8^+^ T cells 363 genes were above quantification threshold in all samples and were included in the analysis. In 38 genotype-selected HS, the expression of 55 genes correlated with sIL-2Rα with a p-value ≤ 0.05 (**Table 1**). All correlations had a negative Spearman rho ranging from -0.49 to -0.32.

**Table 1:** The 55 genes in isolated CD8^+^ T cells from healthy subjects (N = 38) with a negative correlation of gene expression with sIL-2Rα.

Gene name	Accession #	Count range	rho (spearman)	*p*
*REPS1*	NM_001128617.2	835 - 2325	-.490	0.002
*IL7R*	NM_002185.2	4011 - 23244	-.487	0.002
*NFKBIA*	NM_020529.1	571 - 2500	-.474	0.003
*CXCR4*	NM_003467.2	1951 - 13156	-.471	0.003
*CC2D1B*	NM_032449.2	29 - 126	-.467	0.003
*ICAM2*	NM_000873.3	392 - 1203	-.453	0.004
*CCND3*	NM_001760.2	1133 - 2382	-.449	0.005
*JAML*	NM_153206.2	330 - 2137	-.442	0.005
*TXNIP*	NM_006472.1	13276 - 38347	-.440	0.006
*ATG16L1*	NM_198890.2	169 - 453	-.436	0.006
*ERCC3*	NM_000122.1	75 - 159	-.431	0.007
*ITK*	NM_005546.3	1677 - 5425	-.433	0.007
*TNFSF8*	NM_001244.3	190 - 1069	-.430	0.007
*SOCS1*	NM_003745.1	129 - 446	-.429	0.007
*ETS1*	NM_005238.3	7859 - 23305	-.423	0.008
*DDX50*	NM_024045.1	295 - 660	-.420	0.009
*CD40LG*	NM_000074.2	42 - 320	-.419	0.009
*HLA-E*	NM_005516.4	7896 - 16583	-.419	0.009
*DOCK9*	NM_001130048.1	368 - 1369	-.417	0.009
*ABL1*	NM_005157.3	96 - 205	-.415	0.01
*APP*	NM_000484.3	60 - 389	-.414	0.01
*LY9*	NM_001033667.1	1024 - 2813	-.413	0.01
*TRIM39*	NM_021253.3	58 - 172	-.406	0.01
*FLT3LG*	NM_001459.3	879 - 2845	-.404	0.01
*LTA*	NM_000595.2	69 - 470	-.398	0.01
*TNFRSF14*	NM_003820.2	506 - 1252	-.398	0.01
*IL17RA*	NM_014339.6	237 - 699	-.393	0.02
*IL1RAP*	NM_002182.2	57 - 165	-.384	0.02
*TAB1*	NM_153497.2	139 - 409	-.372	0.02
*ICAM3*	NM_002162.3	1845 - 3714	-.371	0.02
*IRAK4*	NM_016123.1	190 - 466	-.372	0.02
*AKT3*	NM_181690.1	614 - 1663	-.370	0.02
*TNFSF4*	NM_003326.2	24 - 99	-.369	0.02
*CD46*	NM_172350.1	1009 - 2292	-.364	0.02
*IL23A*	NM_016584.2	37 - 222	-.362	0.03
*MTMR14*	NM_022485.3	379 - 727	-.356	0.03
*KLRB1*	NM_002258.2	949 - 6280	-.355	0.03
*SELPLG*	NM_001206609.1	1447 - 2757	-.353	0.03
*KLRC1*	NM_002259.3	63 - 530	-.353	0.03
*NFATC1*	NM_172389.1	111 - 248	-.349	0.03
*CD96*	NM_005816.4	1172 - 3729	-.348	0.03
*ABCB1*	NM_000927.3	209 - 664	-.347	0.03
*PSEN1*	NM_000021.2	225 - 553	-.346	0.03
*PNMA1*	NM_006029.4	128 - 362	-.346	0.03
*ZNF346*	NM_012279.2	42 - 165	-.345	0.03
*RPS6*	NM_001010.2	27882 - 111304	-.344	0.03
*CR1*	NM_000651.4	54 - 691	-.339	0.04
*DPP4*	NM_001935.3	100 - 410	-.337	0.04
*BMI1*	NM_005180.5	247 - 510	-.336	0.04
*PPIA*	NM_021130.2	347 - 771	-.336	0.04
*CD44*	NM_001001392.1	3002 - 7065	-.332	0.04
*MFGE8*	NM_001114614.1	79 - 381	-.331	0.04
*IFNGR1*	NM_000416.1	282 - 906	-.329	0.04
*PIK3CD*	NM_005026.3	1145 - 3063	-.329	0.04
*ATG5*	NM_004849.2	241 - 472	-.322	0.05

All correlations had a p-value ≤ 0.05. Data analysis was performed using a Spearman rank correlation. Only genes with mRNA measurements above quantitative threshold in all samples were included.

### Pathway Analysis of Genes Correlating With sIL-2Rα

Using the DAVID Gene Functional Annotation Clustering to identify biological functions of the 55 genes, we identified eight clusters. Among the annotation clusters with the highest enrichment scores were Annotation Cluster 1 (Enrichment Score: 4.36) involving tumor necrosis factor (TNF) (p = 0.00002, q = 0.002), TNF conserved site (p = 0.000005, q = 0.0009) and TNF receptor binding (p = 0.0001, q = 0.005) as well as Annotation Cluster 3 (Enrichment score: 3.43) involving positive regulation of IL-10 (p = 0.00005, q = 0.006), positive regulation of memory T cell differentiation (p = 0.0002, q = 0.01) and positive regulation of T cell proliferation (p = 0.0009, q = 0.06).

### Association Between sIL-2Rα and Surface Receptor IL-7Rα

**Figure 2:**
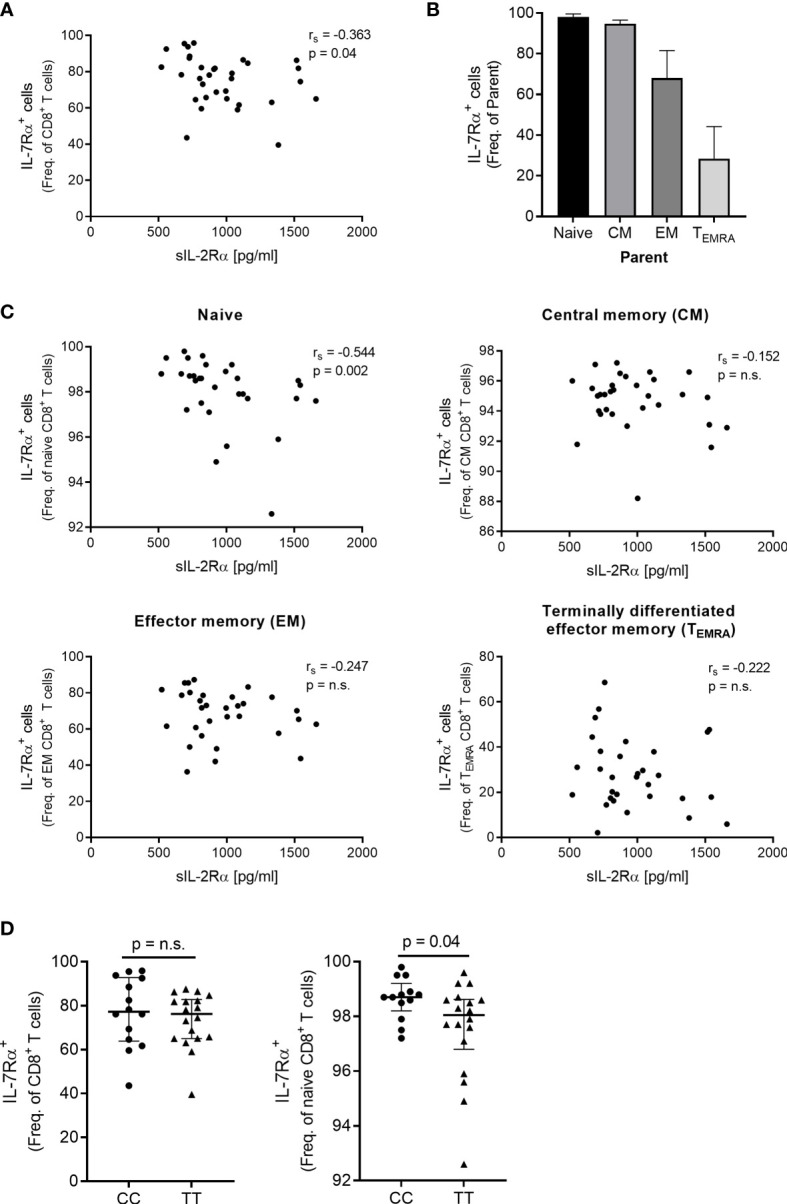
Surface expression of the IL-7Rα on CD8^+^ T cells in relation to sIL-2Rα and the MS-associated SNP rs2104286. **(A)** Spearman correlation between sIL-2Rα and frequency (freq.) of IL-7Rα^+^CD8^+^ T cells in healthy subjects (N = 33). **(B)** The freq. of IL-7Rα^+^ cells on naïve, CM, EM and T_EMRA_ subsets. **(C)** Spearman correlation between sIL-2Rα and the freq. of IL-7Rα^+^ naïve (CD45RA^hi^CCR7^+^) CD8^+^ T cells, freq. of IL-7Rα^+^ central memory (CM, CD45RA^-^CCR7^+^) CD8^+^ T cells, freq. of IL-7Rα^+^ effector memory (EM, CD45RA^-^CCR7^-^) CD8^+^ T cells as well as freq. of IL-7Rα^+^ terminal effector memory (T_EMRA_, CD45RA^hi^CCR7^-^) CD8^+^ T cells in healthy subjects (N = 31). Gating strategy is presented in **Supplementary Figure 2**. **(D)** The MS-associated SNP rs2104286 in relation to the freq. of IL-7Rα^+^CD8^+^ T cells (TT = 18, CC = 14) and the freq. of IL-7Rα^+^ naïve CD8^+^ T cells (TT = 18, CC = 13) in genotype-selected healthy subjects. Genotype comparisons were analysed by a non-parametric Mann-Whitney U test. Error-bars represent median with interquartile range. ns, non-significant.

As we found that *IL7R* that encodes the IL-7 receptor α (IL-7Rα) was among the genes that negatively correlated with sIL-2Rα we investigated by flow cytometry if this association was also present at the protein level in freshly isolated PBMC from 33 genotype-selected HS (see **Supplementary Figure 2** for gating strategy). We observed that sIL-2Rα correlated negatively with the frequency of IL-7Rα^+^CD8^+^ T cells (**Figure 2A**) and found that sIL-2Rα correlated negatively with the frequency of IL-7Rα^+^ naïve CD8^+^ T cells but not with the frequency of IL-7Rα^+^ memory subsets (**Figures 2B, C**). In addition, the frequency of IL-7Rα^+^ naïve CD8^+^ T cells was lower in homozygous risk allele carriers (T) compared to protective allele carriers (C) for the MS-associated *IL2RA* SNP rs2104286 (**Figure 2D**).

## Discussion

In the present study, we explored the effects of the MS-associated *IL2RA* SNP rs2104286 and sIL-2Rα in isolated CD8^+^ T cells, as CD8^+^ T cells are believed to be implicated in MS pathogenesis and IL-2 receptor signalling is crucial for their function.

In HS, we are to our knowledge the first to observe that the CpG-site CpG10:6057082 perturbed by the MS-associated *IL2RA* SNP rs2104286 is allele specific methylated in CD8^+^ T cells. Previous studies that focused on methylation in the *IL2RA* gene did not analyse allele specific methylation at the CpG-site perturbed by the rs2104286 SNP (13, 36). In RRMS patients we observe the same trend, but others need to confirm this as our study included only 12 RRMS patients and only 1 was homozygous carrier of the protective allele (C) for rs2104286.

However, in the isolated CD8^+^ T cells from HS we observed no significant associations between the MS-associated *IL2RA* SNP rs2104286 and changes in methylation of selected CpG-sites in the *IL2RA* promotor and 5’UTR. Furthermore, we only observed few significant associations between expression of immune relevant genes and the rs2104286 SNP, including no association with expression of the *IL2RA* gene. In these analyses, we only included equal sized groups of homozygous carriers of the risk (T) or protective (C) allele as previous genotype-phenotype studies of the rs2104286 SNP have shown a gene-dosage effect (30).

In our study, the number of HS included was based on previous studies of the MS-associated *IL2RA* SNP in CD4^+^ T cells and innate immune cells (18, 30, 37, 38). In these studies, several significant differences (p ≤ 0.05) were identified in cohorts of HS containing approximately the same or fewer homozygous carriers of the protective allele (C) for SNP rs2104286 compared to our study (18, 30, 37, 38). It is possible that a larger cohort of HS would be able to detect differences in *IL2RA* methylation and expression of immune relevant genes in CD8^+^ T cells. However, our findings suggest that in resting CD8^+^ T cells, associations between the SNP rs2104286 and *IL2RA* promotor and 5’UTR methylation as well as expression of immune relevant genes are either not present or smaller than associations previously observed in CD4^+^ T cells and innate immune cells (18, 30, 37, 38). Furthermore, it underlines previous observations that phenotype associations with the MS-associated *IL2RA* SNP rs2104286 are cell-type specific (29, 37), why genotype-phenotype studies need to have a cell-type specific approach.

Our study of association between *IL2RA* methylation and the MS-associated *IL2RA* SNP rs2104286 is based on the results from two previous studies that found two CpG-sites in the *IL2RA* promotor and 5’UTR to be differentially methylated in either MS or T1D compared to HS (13, 36). However, both studies were in a mixed cell population of PBMC (13, 36). Previously, the MS- and T1D-associated CpG-sites in the *IL2RA* 5’UTR and promotor were shown to be methylated in a cell-specific manner and it is possible that the CpG-sites are associated with the MS-associated *IL2RA* SNP rs2104286 in other cell types than CD8^+^ T cells (13, 36). In addition, we only analysed methylation of selected CpG-sites, so other CpG-sites in the *IL2RA* 5’UTR and promotor region of CD8^+^ T cells may be associated with the MS-associated *IL2RA* SNP rs2104286.

Contrary to our observation of few differentially expressed immune relevant genes in relation to the rs2104286 genotype groups, we observed that the expression of several immune relevant genes in CD8^+^ T cells correlated negatively with sIL-2Rα. A previous study estimated that between 15-18% of the variance of sIL-2Rα is explained by the rs2104286 SNP (21). Our findings imply no direct association between expression of immune relevant genes in CD8^+^ T cells and the rs2104286 SNP. However, it does imply an indirect association between the MS-associated SNP rs2104286 and gene expression in CD8^+^ T cells mediated through the effect of the rs2104286 SNP on the sIL-2Rα level. It is possible that sIL-2Rα affects gene expression in resting CD8^+^ T cells through modulation of IL-2 signalling as previous studies have observed that sIL-2Rα antagonizes IL-2 signalling (21, 22). In support of this, we observed that the IL-2-induced genes *SOCS1* (rho = -0.43) and *LTA* (rho = -0.39) were among the genes that negatively correlated with sIL-2Rα in resting CD8^+^ T cells (23, 39). Functional studies are, however, needed to confirm this.

Another gene that negatively correlated with the sIL-2Rα was *IL7R* that encodes the IL-7Rα, an important receptor for the function of CD8^+^ T cells. Also, by use of flow cytometry we observed a negative correlation between sIL-2Rα and the IL-7Rα protein on naïve CD8^+^ T cells. However, IL-2 has been shown to negatively regulate expression of IL-7Rα in CD8^+^ T cells (40), why we had expected a positive correlation between the IL-7Rα and sIL-2Rα as the additional findings in this study imply an inhibition of the CD8^+^ T cells’ IL-2 signalling. It seems unlikely that the negative correlation between sIL-2Rα and IL-7Rα could be due to outliers in our study as the correlation was observed on both a transcription and protein level as well as that *ETS-1* (a positive regulator of IL-7Rα expression) was among the genes that negatively correlated with sIL-2Rα (41).

In MS patients CD8^+^ T cells are proliferating and differentiated towards a memory phenotype and have an increased surface expression of IL-7Rα (2, 8, 42). Unexpected, we observed a lower frequency of IL-7Rα^+^ CD8^+^ T cells in carriers of risk genotype for the MS-associated SNP rs2104286 and an inverse correlation between sIL-2Rα and expression of genes associated with proliferation and memory differentiation. Collectively, our observations suggest that the MS-associated *IL2RA* SNP rs2104286 and the increased sIL-2Rα level associated with the risk rs2104286 genotype do not contribute to the CD8^+^ T cell response observed in MS and possibly abrogate it.

In CD8^+^ T cells *IL2RA* expression is higher and more sustained in an inflammatory compared to a non-inflammatory milieu (25), why our findings in resting CD8^+^ T cells cannot be transferred to activated CD8^+^ T cells. It is possible that during CD8^+^ T cell activation, the MS-associated *IL2RA* SNP rs2104286 and sIL-2Rα have a different impact on the CD8^+^ T cell response in relation to methylation, gene and protein expression. Interestingly, in the same study observing that sIL-2Rα antagonized IL-2 it was also observed that the presence of sIL-2Rα during anti-CD3 stimulation markedly increased the proliferation of T cells (21), a process normally promoted by IL-2 (14). Therefore, studies focusing on sIL-2Rα or the MS-associated *IL2RA* SNP rs2104286 in CD8^+^ T cells during activation are warranted.

In our study results from the different applied methods are consistent and also concordant with previous observations that observed no associations between the SNP rs2104286 and protein or cellular changes in CD8^+^ T cells (29, 30). However, a technical validation of the Nanostring gene expression measurements was not performed. Several studies have observed good correlations between Nanostring and qPCR or microarray gene expression measurements, even though some inconsistencies have been observed when measuring different isoform expressions though this is a general issue in relation to gene expression analysis (43–46). Finally, findings in our study would have benefitted from a validation in a secondary cohort of genotype-selected healthy subjects but due to low frequencies of homozygous carriers of the protective allele (C) for SNP rs2104286 this was not a possibility as described in a previous study by our group (29).

Taken together, our study explored associations of the MS-associated *IL2RA* SNP rs2104286 as well as sIL-2Rα in isolated CD8^+^ T cells. We observed an allele specific methylation at the CpG-site perturbed by the rs2104286 SNP but otherwise found no association between the rs2104286 SNP and methylation of selected CpG-sites in the *IL2RA* gene. Furthermore, our study of gene expression in resting CD8^+^ T cells indicated that the MS-associated *IL2RA* SNP rs2104286 and sIL-2Rα had either minor effect or might negatively regulate the CD8^+^ T cell response - findings that are opposite to those previously observed in CD8^+^ T cells from MS patients. However, our study was in resting CD8^+^ T cells and the results cannot be transferred to activated CD8^+^ T cells. Finally, the study highlighted the importance of a cell-specific approach in the study of MS-associated SNPs.

## Data Availability Statement

The raw data supporting the conclusions of this article will be made available by the authors, subject to the General Data Protection Regulation (GDPR) in the European Union.

## Ethics Statement

The studies involving human participants were reviewed and approved by the ethical committee in the Capital Region (KF-01 314009, KF-01114309). The patients/participants provided their written informed consent to participate in this study.

## Author Contributions

SB: Conceptualization, methodology design and development, data collection, performing experiments, data and statistical analysis, writing – original first draft. H-ML: Conceptualization, methodology design and development, performing experiments, data and statistical analysis, writing – reviewing and editing. ME: Data collection, methodology design and development, writing – reviewing and editing. HU: Data collection, writing – reviewing and editing. AO: Data collection, writing – reviewing and editing. FS: Conceptualization, methodology design and development, supervision, writing – reviewing and editing. HS: Conceptualization, methodology design and development, performing experiments, data and statistical analysis, supervision, writing – reviewing and editing. All authors contributed to the article and approved the submitted version.

## Funding

This research was supported by the Danish Multiple Sclerosis Society, the Foundation for Research in Neurology, Johnsen og Hustru’s Mindelegat, Fonden til Lægevidenskabens Fremme, A.P. Møller og Hustru Chastine Mc-Kinney Møllers Fond til Almene Formaal and Novartis Denmark.

## Conflict of Interest

SB has received support for congress participation from Biogen and Merck. H-ML currently works at AGC Biologics but had no conflicts of interest at the time of performing the study described in this paper. AO has served on scientific advisory boards for Biogen Idec, Novartis, and Sanofi Genzyme; has received research support from Novartis and Biogen Idec; has received speaker honoraria from Biogen Idec, Novartis and TEVA; and has received support for congress participation from Merck, TEVA, Biogen, Roche, Novartis, and Sanofi Genzyme. FS has served on scientific advisory boards for, served as consultant for, received support for congress participation or received speaker honoraria from Alexion, Biogen, Merck, Novartis, Roche, and Sanofi Genzyme. His laboratory has received research support from Biogen, Merck, Novartis, Roche, and Sanofi Genzyme.

The remaining authors declare that the research was conducted in the absence of any commercial or financial relationships that could be construed as a potential conflict of interest.

## Publisher’s Note

All claims expressed in this article are solely those of the authors and do not necessarily represent those of their affiliated organizations, or those of the publisher, the editors and the reviewers. Any product that may be evaluated in this article, or claim that may be made by its manufacturer, is not guaranteed or endorsed by the publisher.

## Supplementary Material

Supplementary Figure 1 Association between the MS-associated SNP rs2104286 and methylation of the 5 CpG-sites in the 5’UTR and promotor of the *IL2RA* gene in genotype-selected heathy subjects (CC = 20; TT = 20). Data were analysed by an unpaired t-test. Error-bars represent mean with SD.

Supplementary Figure 2 Gating strategy for the IL-7Rα and CD8^+^ T cell differentiation analysis. **(A)** Gating of CD8^+^ T cells. **(B)** On CD8^+^ T cells, differential expression of CD45RA and CCR7 was used to define 4 subsets: Naïve (CD45RA^hi^CCR7^+^), central memory (CM, CD45RA-CCR7^+^), effector memory (EM, CD45RA^-^CCR7^-^) and terminally differentiated effector memory (T_EMRA_, CD45RA^hi^CCR7^-^). Naïve and T_EMRA_ were visually gated while EM and CM were gated based on matched isotype controls for CD45RA and CCR7. **(C)** Matched isotype control for CD45RA and CCR7 on CD8^+^ T cells. **(D)** Matched isotype control for IL-7Rα. **(E)** Gating of IL-7Rα^+^ cells on CD8^+^ T cells that was performed by use of matched isotype control for IL-7Rα (CD127). The gating was subsequently applied to the four CD8^+ ^T cell subsets.
