# Supplementary figures and images for: IL2RA Methylation and Gene Expression in Relation to the Multiple Sclerosis-Associated Gene Variant rs2104286 and Soluble IL-2Rα in CD8+ T Cells
Source: Front Immunol. 2021 Jul 27;12:676141. doi: 10.3389/fimmu.2021.676141 (PMC8353370; doi:10.3389/fimmu.2021.676141)

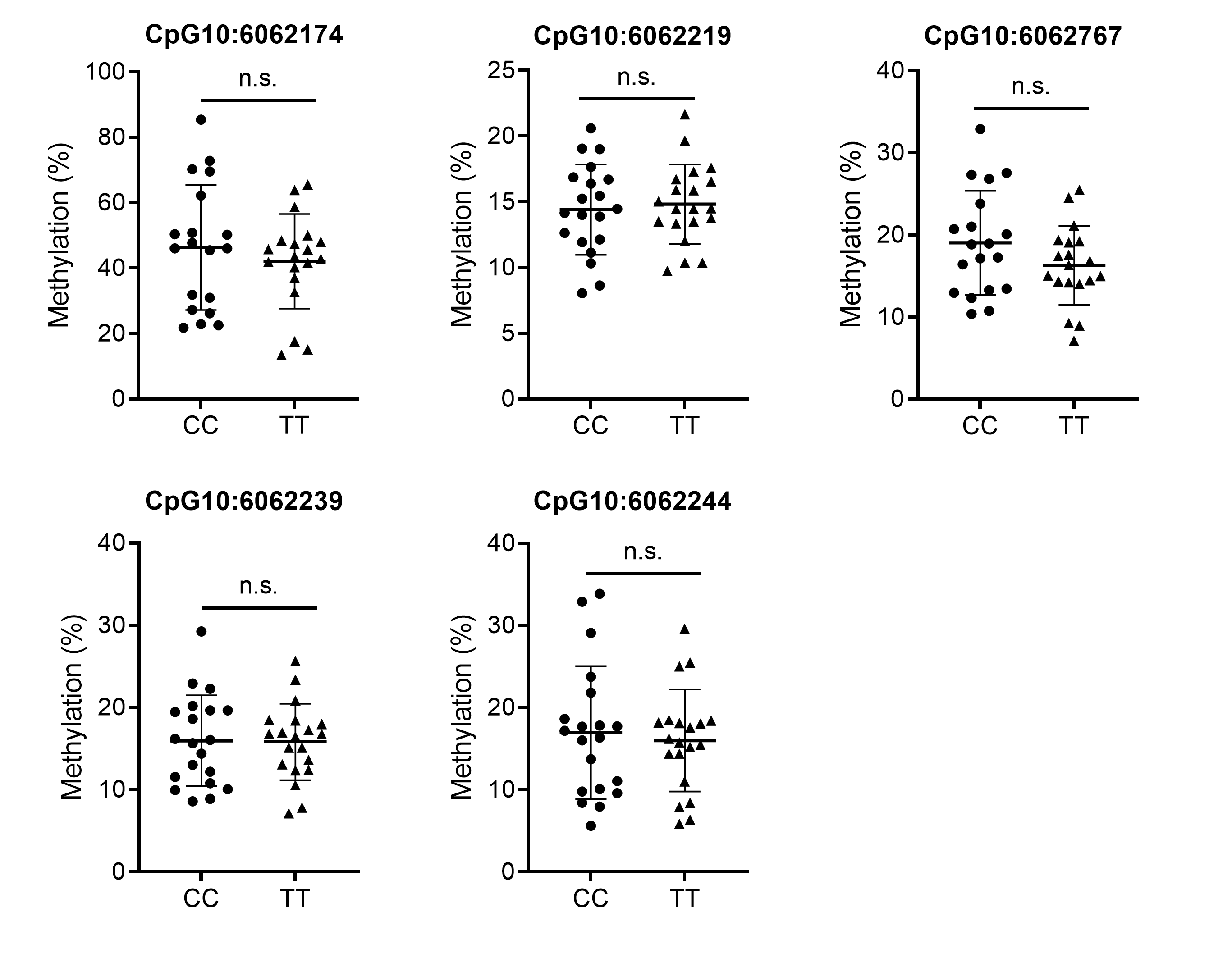

Supplement: Supplementary Figure 1 — Association between the MS-associated SNP rs2104286 and methylation of the 5 CpG-sites in the 5’UTR and promotor of the IL2RA gene in genotype-selected heathy subjects (CC = 20; TT = 20). Data were analysed by an unpaired t-test. Error-bars represent mean with SD. [file Image_1.tif]

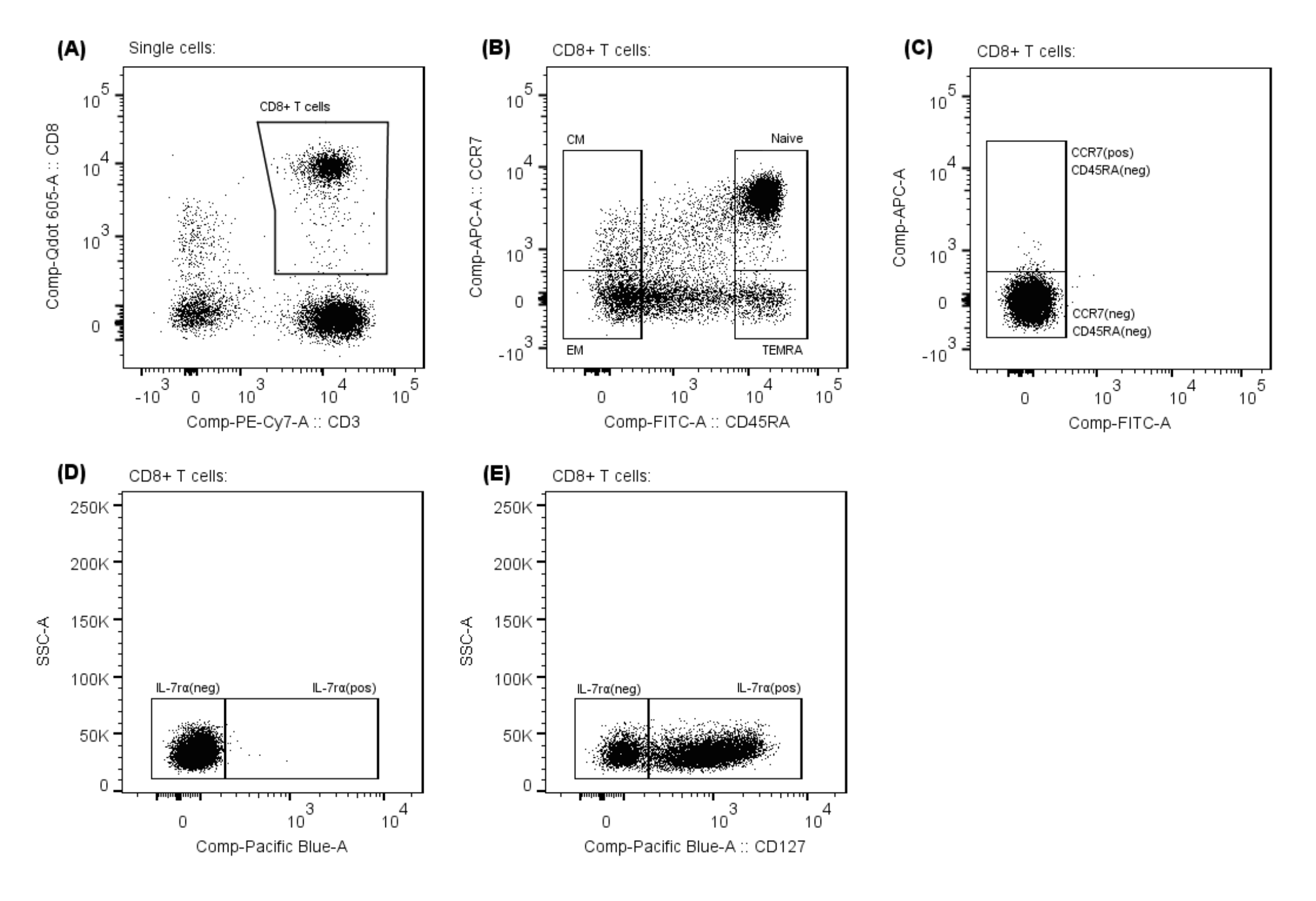

Supplement: Supplementary Figure 2 — Gating strategy for the IL-7Rα and CD8+ T cell differentiation analysis. (A) Gating of CD8+ T cells. (B) On CD8+ T cells, differential expression of CD45RA and CCR7 was used to define 4 subsets: Naïve (CD45RAhiCCR7+), central memory (CM, CD45RA-CCR7+), effector memory (EM, CD45RA-CCR7-) and terminally differentiated effector memory (TEMRA, CD45RAhiCCR7-). Naïve and TEMRA were visually gated while EM and CM were gated based on matched isotype controls for CD45RA and CCR7. (C) Matched isotype control for CD45RA and CCR7 on CD8+ T cells. (D) Matched isotype control for IL-7Rα. (E) Gating of IL-7Rα+ cells on CD8+ T cells that was performed by use of matched isotype control for IL-7Rα (CD127). The gating was subsequently applied to the four CD8+ T cell subsets. [file Image_2.tif]
