# Supplementary material for: IL2RA Methylation and Gene Expression in Relation to the Multiple Sclerosis-Associated Gene Variant rs2104286 and Soluble IL-2Rα in CD8+ T Cells
Source: Front Immunol. 2021 Jul 27;12:676141. doi: 10.3389/fimmu.2021.676141 (PMC8353370; doi:10.3389/fimmu.2021.676141)
Supplement: Supplementary file 4 [file DataSheet_2.docx]

## Supplementary methods: DNA extraction protocol – cohort 2

The protocol is based on the instruction manual for *“User-Developed Protocol”* provided by *QIAGEN* (https://www.qiagen.com/pt/resources/faq?id=54b752f7-c429-41f5-bdcd-c27263ae0255). The protocol is optimized and thus differs from the online available protocol. DNA from cohort 2 have been purified using this protocol.

### Notes

- This protocol is for samples lysed in 700ul QIAzol^TM^ lysis reagent. Chloroform separation and RNA purification were carried out prior to this protocol. All the aqueous phase must be removed from the interphase and phenol phase. DNA purification is carried out within 24 hr. after RNA purification.
- You can vortex the samples numerous times along the way as long as you only vortex shortly ≤5s.
- Unless stated otherwise, all protocol steps should be performed at room temperature (15-25˚C).

### Material

Solutions

- 100% Ethanol
- 75% Ethanol
- 0.1M Sodium citrate solution (0.1M sodium citrate in 10% Ethanol)
- TE buffer (10mM Tris pH 8.0, 1mMEDTA pH 8.0)

Equipment

- Nanodrop 2000 spectrophotometer (by *Thermo-Scientific*)
- Centrifuge
- Vortexer
- Heating block

Consumables

- 1.5mL DNA,RNA, RNase free Eppendorf tubes
- 2.0 mL DNA,RNA, RNase free Eppendorf tubes

### DNA Precipitation

- Remove any residues of aqueous phase.
- Add 300µl of 100% Ethanol to the interphase and phenol phase. Try to put the Ethanol very gently on top of the interphase if possible. Mix gently by inversion. If the interphase can be intact it would be good. If you are not able to see the interphase as and intact layer after gently mixing than vortex shortly to mix the Ethanol with the interphase and organic phase.
- Incubate samples at room temp. (15-15˚C) for 2-3 min.
- Centrifuge at 2000-2500g for 2 min at 4˚C to sediment the DNA.
- Remove the phenol/ethanol supernatant completely if possible but be careful not to remove the pellet. The pellet is very loose.
  - To make sure you do not discard the pellet pipette the supernatant in a new clean eppendorf tube and shake it to see if any pellet is in it. If so, try to fish it out.

### Washing

- Add 1mL citrate solution to the DNA pellet.
- Add 1mL citrate solution to 2 new 1.5mL eppendorf tubes for each sample (e.g. for 10 samples prepare 40 clean eppendorf tubes with 1mL citrate solution in each).
- Now, try if you can “fish” out the pellet from the old tube with a 1000ul pipette tip. Aspirate the pellet to locate it but try not to suck it up in the pipette but let it stick to the opening of the tip so it remain in one piece. Place the pellet into the first new 1.5mL eppendorf tube with wash solution in. fish up the pellet again and place it in the second new 1.5mL eppendorf tube.
  - If the pellet is in more pieces try to fish them all up.
  - This is a critical step in the protocol, because the phenol is a major contaminator and most of the phenol is sticking to the sides of the eppendorf tube.
- Incubate the pellet in for 5-30 min and invert it now and then if possible (inversion is not necessary)
  - Always make sure that your pellet stays within the tube you are working with. The pellet should be visible.
- Centrifuge at 2000g for 5 min. at 4˚C and remove the supernatant and add 1mL wash solution again. If you have time leave the pellet in the wash solution for 5-30min. invert the tube now and then (inversion is not necessary).
- Remove the supernatant and wash the pellet in 1-1.5mL 75% ethanol. Incubate for 5-20 min and mix by inversion now and then.
  - After the wash steps the undissolved DNA pellet can be stored in 75% Ethanol at 4˚C for over 3 month.
- Centrifuge at 2000g for 5min. At 4˚C.
- Remove ALL the Ethanol from the sample
  - Pipet as much as you can and leave your sample approx. 3 min. with led open in a 37˚C preheated heating block.
  - Place the heating block under the ventilator.

### Elution

- Dissolve your pellet in 60ul TE buffer.
  - This can be difficult, therefore, place your samples directly onto a 70˚C pre-heated heating block for 20-25 min and vortex shortly (≤5s) afterwards or every 5 min during heating.
  - If the pellet is very big add 10-20uL TE buffer more
- Store your DNA samples at -80˚C
